# Folate Pathway Polymorphisms and Tadalafil Response in Men with Diabetes Mellitus and Erectile Dysfunction: An Exploratory Study

**DOI:** 10.3390/jcm15197349

**Published:** 2026-09-22

**Authors:** Boštjan Hostnik, Andrej Janež, Katja Goričar, Tanja Blagus, Vita Dolžan, Andrej Kastrin, Jasna Klen

**Affiliations:** 1 Division of Internal Medicine, Department of Endocrinology, Diabetes and Metabolic Diseases, University Medical Centre Ljubljana, 1000 Ljubljana, Slovenia; hostnikb@gmail.com (B.H.); andrej.janez@kclj.si (A.J.); 2 Faculty of Medicine, University of Ljubljana, 1000 Ljubljana, Slovenia; 3 Pharmacogenetics Laboratory, Institute of Biochemistry and Molecular Genetics, Faculty of Medicine, University of Ljubljana, 1000 Ljubljana, Slovenia; katja.goricar@mf.uni-lj.si (K.G.); tanja.blagus@mf.uni-lj.si (T.B.); vita.dolzan@mf.uni-lj.si (V.D.); 4 Institute for Biostatistics and Medical Informatics, Faculty of Medicine, University of Ljubljana, 1000 Ljubljana, Slovenia; andrej.kastrin@mf.uni-lj.si; 5 Division of Surgery, Department of Abdominal Surgery, University Medical Centre Ljubljana, 1000 Ljubljana, Slovenia

**Keywords:** PDE5 inhibitors, pharmacogenetics, folate metabolism, *MTHFR*, *MTR*, endothelial dysfunction, single-nucleotide polymorphism

## Abstract

**Background/Objectives:** Erectile dysfunction (ED) is a common complication of diabetes mellitus (DM), largely driven by endothelial dysfunction and impaired nitric oxide signaling. Although phosphodiesterase type 5 inhibitors (PDE5i) such as tadalafil are effective, treatment response varies considerably between individuals. Genetic variation in the folate–homocysteine pathway may influence endothelial function and contribute to this variability. To investigate the association of selected polymorphisms in folate–homocysteine pathway genes (*MTHFR* rs1801133 and rs1801131, *MTR* rs1805087 and *MTRR* rs1801394) with ED severity and response to tadalafil treatment in men with DM. **Methods:** In this prospective exploratory pharmacogenetic study, 78 men with DM and ED were treated with tadalafil 5 mg once daily for three months. ED was assessed using the International Index of Erectile Function (IIEF-5) questionnaire before and after treatment. The pre-specified primary endpoint was treatment response, defined as a ≥4-point increase in IIEF-5 score (based on the minimal clinically important difference for the IIEF erectile function domain). Genotyping was performed using competitive allele-specific PCR. Associations between genetic variants and treatment outcomes were evaluated using logistic regression. **Results:** Before treatment 24 patients (30.8%) had moderate or severe ED. No significant associations were observed between the investigated polymorphisms and baseline ED severity. IIEF-5 scores significantly increased during the three-month tadalafil treatment period, with 80.8% of patients showing improvement and 60.3% achieving a clinically relevant increase of ≥4 points in IIEF-5 score. None of the investigated polymorphisms was associated with the primary endpoint after Bonferroni correction across the three global genotype tests. In the secondary ‘any improvement’ analysis, a nominal association was observed for MTR rs1805087 in a small subgroup (*n* = 5 GG carriers; unadjusted OR = 0.15, 95% CI 0.02–0.89; *p* = 0.037), which was attenuated after multivariable adjustment (OR = 0.16, 95% CI 0.02–1.08, *p* = 0.060) and is regarded as hypothesis-generating only. **Conclusions:** Genetic variation in the folate pathway does not appear to influence baseline ED severity or tadalafil responsiveness in men with DM, although this exploratory study was not powered to exclude modest genetic effects. These preliminary findings require confirmation in larger studies that also incorporate plasma homocysteine and B-vitamin measurement.

## 1. Introduction

Diabetes mellitus (DM) is a major global health burden, with prevalence projected to reach 853 million adults worldwide by 2050 [1]. Erectile dysfunction (ED) is one of its most common complications, affecting more than half of men with diabetes and occurring earlier and with greater severity than in the general population. Beyond its impact on quality of life, ED reflects generalized vascular and endothelial dysfunction and may serve as an early marker of cardiovascular diseases, which represent a major concern in diabetic patients with ED [2].

The pathophysiology of diabetic ED is multifactorial, involving endothelial dysfunction, oxidative stress, autonomic neuropathy, hormonal disturbances, and structural changes in cavernosal tissue. A central mechanism is impaired nitric oxide (NO) signaling, which reduces smooth muscle relaxation and penile blood flow [3,4,5].

Phosphodiesterase type 5 inhibitors (PDE5i), including tadalafil, enhance erectile function by inhibiting cGMP degradation and amplifying residual NO-mediated signaling [5]. However, their efficacy depends not only on sufficient upstream NO production, but also on preserved cavernosal structure and endothelial integrity. In diabetic patients, response rates to PDE5i are lower than in the general ED population [2,6], and variability in treatment outcomes may reflect differences in endothelial integrity, structural remodeling and potentially genetic background [7,8].

One pathway of particular interest in diabetic patients with ED is the folate–homocysteine metabolism. It has been hypothesized that disturbances in this pathway can result in hyperhomocysteinemia, which has been linked to endothelial dysfunction, oxidative stress and impaired erectile function [9,10]. The proposed pathophysiological mechanism involves homocysteine-mediated oxidation of tetrahydrobiopterin (BH4), an essential cofactor for endothelial nitric oxide synthase (eNOS). BH4 depletion promotes eNOS uncoupling, leading to the generation of superoxide rather than nitric oxide (NO), thereby reducing NO bioavailability and compromising the cGMP-dependent smooth muscle relaxation required for penile erection [11]. Consistent with this mechanism, elevated homocysteine levels and lower folate concentrations have been associated with ED severity in several observational studies [12].

Key enzymes in this pathway include methylenetetrahydrofolate reductase (MTHFR), methionine synthase (MTR), and methionine synthase reductase (MTRR). Functional polymorphisms in the corresponding genes have been associated with altered enzyme activity and homocysteine metabolism. Specifically, the *MTHFR* rs1801133 (c.677C>T; p.Ala222Val) variant decreases MTHFR thermostability and enzymatic activity, with homozygous carriers showing approximately 30–50% of wild-type enzymatic activity [13]. This variant has also been consistently associated with altered homocysteine concentrations, particularly in the context of low folate status [14]. Associations between *MTHFR* C677T and vascular phenotypes have also been reported, although these findings have varied across populations and vascular outcomes.

The *MTHFR* rs1801131 (c.1298A>C; p.Glu429Ala) variant has also been shown to influence folate metabolism and may interact with rs1801133 to modulate homocysteine levels. In the remethylation cycle, the *MTR* rs1805087 (c.2756A>G; p.Asp919Gly) variant may impair methionine synthase activity [15], thereby reducing the conversion of homocysteine to methionine. Finally, the *MTRR* rs1801394 (c.66A>G) variant affects the regeneration of methionine synthase cofactor activity and has been linked to altered homocysteine concentrations in some populations [16]. These metabolic alterations may contribute to endothelial dysfunction and reduced NO bioavailability.

We hypothesized that genetic variation in the folate–homocysteine pathway could influence both ED severity and responsiveness to PDE5i therapy by modulating upstream NO production. However, this proposed mechanistic cascade from genotype to altered homocysteine metabolism, endothelial dysfunction, and finally PDE5i response remains biologically plausible but has not been directly validated in the context of erectile dysfunction. Importantly, the present study was designed as an exploratory pharmacogenetic association study and did not include direct measurements of plasma homocysteine, endothelial function, or NO-related biomarkers. Despite strong biological plausibility, evidence linking folate-pathway genetic variants to ED or response to PDE5i remains limited and inconsistent. Most pharmacogenetic studies of PDE5i response have focused on genes directly involved in penile NO signaling (e.g., endothelial nitric oxide synthase [NOS3]) or on PDE5A itself [7,17]. In contrast, investigations of upstream metabolic pathways that regulate NO bioavailability, such as the folate–homocysteine cycle, are insufficient, and data regarding their influence on PDE5i response are particularly limited.

The aim of this prospective exploratory pharmacogenetic study was to investigate the association of selected polymorphisms in the *MTHFR*, *MTR* and *MTRR* genes with ED severity and response to tadalafil treatment in men with DM, thereby extending pharmacogenetic research from the drug target and direct signaling pathways toward upstream metabolic regulation. To our knowledge, this is among the first studies to examine folate-pathway genes, upstream of the classical NO-synthesis and PDE5-signaling genes that have dominated prior pharmacogenetic research in ED [7], in relation to PDE5i response specifically in men with DM; this upstream, metabolic-pathway perspective is the principal novelty of the present study.

## 2. Materials and Methods

### 2.1. Study Design

This prospective genetic association study included patients with a diagnosis of type 1 DM or type 2 DM and concomitant ED. All participants were assessed using standardized protocols for clinical evaluation, data collection, genotyping and statistical analysis.

The primary endpoint was treatment response, defined as a ≥4-point increase in IIEF-5 score (Section 2.2); baseline ED severity, absolute IIEF-5 score change, and ‘any improvement’ were evaluated as secondary/exploratory outcomes. The study was conducted in accordance with the Declaration of Helsinki and the Slovenian Code of Medical Ethics and received ethical approval from the National Medical Ethics Committee of the Republic of Slovenia at the Ministry of Health (KME 0120-337/2023/3).

### 2.2. Study Participants

A total of 78 participants with clinical diagnosis of DM and ED were enrolled in the study. Participants were recruited from University Medical Centre Ljubljana and Health Centre Kočevje between May 2024 and March 2025. Inclusion criteria were confirmed diagnosis of DM and ED (participants who scored 21 points or less on the International Index of Erectile Function (IIEF-5) questionnaire), age between 40 and 65 years and signed informed consent. ED was classified into five severity grades: absence of ED (IIEF-5 score 22–25), mild (17–21), mild to moderate (12–16), moderate (8–11) and severe (1–7) [18].

Exclusion criteria included treatment with organic nitrates, a history of cardiovascular events, prostate malignancy, psychogenic ED, premalignant or malignant genital lesions, Peyronie’s disease, congenital penile disorders, previous pelvic injury, surgery or radiotherapy and a prostate-specific antigen (PSA) level ≥ 3.0 μg/L.

All participants received treatment with tadalafil 5 mg daily for a duration of three months. Information on prior exposure to PDE5 inhibitors and on adherence to the prescribed tadalafil regimen was not systematically collected in this study; this is addressed as a limitation in the Discussion.

Three outcome measures were used to characterize treatment response and are reported separately throughout this manuscript to avoid conflating statistical and clinical significance: (1.) any numerical increase in IIEF-5 score from baseline (‘any improvement’), a directional measure of change; (2.) an increase of at least 4 points in IIEF-5 score, the pre-specified primary endpoint of this study and based on the minimal clinically important difference (MCID) established by anchor-based analyses for the IIEF erectile function domain [18], applied here to the abbreviated IIEF-5 (see limitations in the Discussion); and (3.) a post-treatment IIEF-5 score ≥ 22, indicating categorical resolution of ED symptoms. Participants meeting criterion (2.) were considered responders for the primary analysis. Serum folate concentration was measured before treatment using the CIA immunoassay method according to standard clinical practice, with reference values between 6 and 39 nmol/L.

### 2.3. DNA Isolation and Genotyping

Genomic DNA was extracted from peripheral venous blood collected in EDTA-containing tubes (BD Vacutainer Blood Collection Tube, Becton Dickinson, NJ, USA). DNA isolation was performed using the FlexiGene DNA Kit (Qiagen, Hilden, Germany) according to the manufacturer’s instructions. Polymorphisms in folate–homocysteine pathway genes (*MTHFR*, *MTR*, *MTRR*) were selected based on their minor allele frequencies (MAFs) in a Caucasian population and previously reported functional relevance. Only SNPs with MAF > 0.05 were included in the analysis.

The selected polymorphisms were genotyped using competitive allele-specific PCR (KASP) assays according to the manufacturer’s protocol (LGC Biosearch Technologies, Guildford, UK). To ensure genotyping accuracy, control samples with known genotypes were included on each PCR plate, and approximately 20% of samples were randomly selected for blinded repeat genotyping. Genotyping success rate and concordance among duplicate samples were 100%. Genotype labels reported throughout this manuscript follow the reference (plus/forward) strand orientation given by the HGVS genomic descriptions; where the KASP assay design reports an allele on the complementary strand, genotype letters were converted accordingly to ensure a single, consistent nomenclature.

### 2.4. Statistical Analysis

Continuous variables were presented as medians with interquartile ranges (IQRs), and categorical variables as counts and percentages. The normality of continuous distributions was assessed using the Shapiro–Wilk test; because most variables deviated from normality, non-parametric tests were used: the Mann–Whitney U test for comparisons between two independent groups, the Kruskal–Wallis test for comparisons among more than two groups, and the Wilcoxon signed-rank test for paired samples across timepoints. Categorical variables were compared using the chi-square test, or Fisher’s exact test when expected cell counts were below five. All tests were two-tailed.

Minor allele frequencies (MAFs) were calculated for all genotyped polymorphisms. As a quality-control step performed without reference to treatment outcome, deviation from Hardy–Weinberg equilibrium (HWE) was assessed for each polymorphism using the chi-square test (exact test as confirmation), with a Bonferroni-corrected threshold of *p* < 0.0125 (0.05/4); polymorphisms showing significant deviation were excluded from the outcome analyses.

Associations between polymorphisms and categorical outcomes were evaluated using logistic regression, reporting odds ratios (ORs) with 95% confidence intervals (CIs). Because several genotype subgroups were small and some contingency cells contained zero events, all models were fitted using Firth’s penalized maximum likelihood [19], with profile-penalized-likelihood CIs and penalized likelihood-ratio *p*-values. Unadjusted models included genotype only. Adjusted models of baseline ED severity additionally included HbA1c, age, and duration of DM; adjusted models of treatment response also included baseline IIEF-5 score, because baseline severity strongly influences subsequent change through ceiling effects and regression to the mean. Other potential confounders (e.g., BMI, blood pressure, lipid profile, smoking status, prior PDE5i exposure and adherence) were omitted owing to incomplete documentation.

The primary hypothesis family was pre-specified as one global genotype test per retained polymorphism for the single primary endpoint (≥4-point IIEF-5 increase), with a Bonferroni-corrected threshold of α = 0.05/*k*, where *k* is the number of polymorphisms entering the outcome analyses (Section 3.4). Each global test entered genotype as a three-level factor and compared this model with the same model omitting genotype using a Firth-penalized likelihood-ratio test on 2 degrees of freedom, avoiding assumptions about the mode of inheritance. Genotype-specific ORs and *p*-values are reported as exploratory estimates without multiplicity adjustment. Additive (Kruskal–Wallis) and dominant (Mann–Whitney) genetic models were applied only in exploratory analyses of continuous IIEF-5 change. Baseline ED severity, continuous IIEF-5 change, ‘any improvement’, post-treatment IIEF-5 ≥ 22 and serum folate concentration were pre-specified secondary/exploratory outcomes, for which nominal *p*-values are reported descriptively without confirmatory inference.

Power for the primary endpoint was estimated for the 2-degree-of-freedom global genotype test at the corrected threshold: for a polymorphism with an MAF of 30%, the study had 80% power to detect ORs ≥ 7.7 for a ≥4-point IIEF-5 improvement (secondary outcomes, at nominal α = 0.05: ORs ≥ 4.0 for baseline ED severity, ORs ≥ 5.1 for ‘any improvement’). The study was therefore powered to detect large genetic effects, and modest associations cannot be excluded. Statistical analyses were performed using R (version 4.6.1).

## 3. Results

### 3.1. Patients’ Characteristics

A total of 78 male patients with DM and ED were included in the study. The median age was 58 (53–62.3) years, with the median duration of DM 11 (6–17) years. Most patients had type 2 DM (88.5%). Median HbA1c was 7.2 (6.4–8.3)%. Baseline hormonal and biochemical characteristics are summarized in Table 1. All enrolled patients completed both the baseline and 3-month follow-up IIEF-5 assessments and were included in all primary analyses; genotyping success and duplicate concordance were 100% (Section 2.3). Serum folate concentration was available for 69 of 78 patients (88.5%); missing folate values (*n* = 9) were due to the sample not being drawn at the baseline visit, and these patients were excluded only from the folate-specific analyses (Section 3.5).

ED severity and treatment response data are presented in Table 2. At baseline, the median IIEF-5 score was 14 (11–17). In total, 24 patients (30.8%) had moderate or severe ED before treatment. After three months of treatment with tadalafil, the median IIEF-5 score increased to 19.5 (16–22), corresponding to a median improvement of 4 (2–7) points (Figure 1). Overall, 63 patients (80.8%) demonstrated improvement in IIEF-5 score, while 15 (19.2%) showed no change (*p* < 0.001). Consistent with the single-arm, pre–post design of this study (no placebo or untreated comparison group), this increase is described as a within-group change over the treatment period rather than as direct evidence of a pharmacological treatment effect (see Discussion).

A clinically relevant improvement (≥4-point increase, the pre-specified primary endpoint) was observed in 47 patients (60.3%). In 23 patients (29.5%), post-treatment IIEF-5 scores reached ≥22, indicating resolution of ED symptoms.

### 3.2. Genotype Frequencies

Genotype distributions for the investigated polymorphisms in *MTHFR*, *MTR* and *MTRR* genes are presented in Table 3. MAFs were consistent with those reported in European populations. All polymorphisms were in HWE except for *MTRR* rs1801394, which showed significant deviation (*p* = 0.003) and was therefore excluded from further analysis (possible explanations for this deviation are discussed in Section 4).

### 3.3. Folate Pathway Polymorphisms and Degree of Erectile Dysfunction

No significant associations were observed between the studied polymorphisms and change in IIEF-5 score (Table 4). Analysis of ED severity categories (mild/mild-to-moderate vs. moderate/severe) also showed no significant associations for any of the investigated polymorphisms, even after adjustment for HbA1c, age, and DM duration (Table 5).

### 3.4. Folate Pathway Polymorphisms and Response to Tadalafil Treatment

For the primary endpoint (i.e., a ≥4-point increase in IIEF-5 score), no statistically significant associations were observed for any polymorphism (all global genotype tests *p* > 0.0167, the Bonferroni-corrected threshold of 0.05/3), either unadjusted or after adjustment for HbA1c, age, DM duration, and baseline IIEF-5 score (Table 6). This included the *MTR* rs1805087 GG genotype, for which carriers showed a numerically lower likelihood of achieving the primary ≥4-point response compared with AA carriers, but the association did not approach significance (unadjusted OR = 0.35, 95% CI 0.05–2.01, *p* = 0.235; adjusted OR = 0.46, 95% CI 0.07–2.83, *p* = 0.401).

In the analysis of ‘any improvement’ in IIEF-5 score (Table 7), no statistically significant associations were observed between *MTHFR* polymorphisms (rs1801131 and rs1801133) and treatment response. For *MTR* rs1805087, carriers of the GG genotype (a small subgroup of only five patients) showed a nominal association with a lower likelihood of ‘any improvement’ compared with AA carriers (unadjusted OR = 0.15, 95% CI 0.02–0.89, *p* = 0.037). This association was attenuated after multivariable adjustment (adjusted OR = 0.16, 95% CI 0.02–1.08; *p* = 0.060), suggesting that at least part of the unadjusted signal may reflect differences in included covariates rather than genotype per se. As a pre-specified exploratory outcome analyzed without multiplicity adjustment, this nominal association in a five-patient subgroup should be regarded as hypothesis-generating rather than as evidence of a clinically meaningful genotype effect; the resulting confidence intervals are wide, and the estimate is imprecise.

Baseline clinical characteristics were broadly similar between responders and non-responders (Table 8). A non-significant trend toward lower baseline IIEF-5 scores among responders (median 13.0 vs. 16.0 points; *p* = 0.099) is consistent with a possible ceiling effect, whereby patients with milder baseline dysfunction have less scope to gain ≥4 points regardless of genotype. Diabetes type and SGLT2 inhibitor use did not differ significantly between groups, although type 1 diabetes was numerically more frequent among responders (8/47, 17%) than non-responders (1/31, 3.2%; *p* = 0.079); given the small number of type 1 patients (*n* = 9 overall), this should not be over-interpreted.

### 3.5. Folate Pathway Polymorphisms and Folate Concentrations

Median serum folate concentration before treatment was 26.4 (20.3–30.2) nmol/L. In total, five (7.2%) patients had folate levels above the reference values, while no patient had folate levels below the reference values. No significant associations were observed between the investigated polymorphisms and serum folate concentrations before treatment with tadalafil (all *p* > 0.05).

## 4. Discussion

In this prospective pharmacogenetic study we investigated whether common polymorphisms in folate–homocysteine pathway genes influence ED severity and response to tadalafil therapy. The main finding was the absence of significant associations between the investigated polymorphisms and tadalafil treatment response on the Bonferroni-corrected global genotype tests for the primary endpoint; likewise, no significant associations with baseline ED severity were observed in the secondary analyses.

Because plasma homocysteine, endothelial function, NO-related biomarkers and oxidative stress markers were not measured in this study, our data can test the association between genotype and clinical outcomes but cannot directly validate the proposed mechanistic chain linking folate-pathway genotype to homocysteine levels, endothelial dysfunction, NO bioavailability and, ultimately, tadalafil response. This mechanistic pathway should therefore be regarded as a biologically plausible hypothesis motivating this study, rather than as a mechanism demonstrated by our results.

IIEF-5 scores significantly increased during the three-month tadalafil treatment period in the majority of participants included in our study. As this was a single-arm study without a placebo or untreated comparison group, this increase cannot be unambiguously attributed to a pharmacological effect of tadalafil alone, and should be interpreted with this design limitation in mind. The overall magnitude of improvement observed in our cohort is consistent with previous studies demonstrating that PDE5i remain effective in diabetic patients despite the complex pathophysiology of diabetic ED. In a systematic review and meta-analysis, Kouidrat et al. reported a high prevalence of ED among men with DM and emphasized that treatment outcomes are generally less favorable than in non-diabetic populations [20]. Similarly, Defeudis et al. highlighted that diabetic ED is characterized by a combination of vascular, neurological, endocrine, and structural abnormalities that may reduce responsiveness to PDE5i [2]. These observations are consistent with our finding that although most patients improved following tadalafil treatment, approximately 40% did not achieve a clinically meaningful response, which underscores the need for predictive biomarkers to guide individualized treatment in this population.

There is substantial evidence supporting the role of homocysteine metabolism in endothelial function and erectile physiology. Hyperhomocysteinemia has been identified as an independent risk factor for endothelial dysfunction, promoting oxidative stress, inflammation and reduced NO bioavailability [10,21]. Conversely, lower folate levels have been linked to impaired erectile function, suggesting that disruption of this metabolic pathway may contribute to ED pathogenesis [9,22]. Men with ED had significantly higher homocysteine concentrations and lower folate levels compared with controls [9]. A subsequent meta-analysis further confirmed a significant association between elevated homocysteine levels and ED [12]. These studies suggest that disturbances in folate metabolism may contribute to erectile impairment through adverse effects on vascular function.

However, we did not observe any association between the investigated folate pathway polymorphisms and baseline ED severity. This finding is perhaps unsurprising given the multifactorial nature of diabetic ED. While endothelial dysfunction plays a central role, disease severity is influenced by numerous interacting factors, including age, duration of DM, glycemic control, obesity, hypertension, dyslipidemia, androgen status, neuropathy, medication use, and psychological factors [2].

Recently, Bai et al. evaluated 1254 men attending a fertility clinic and found no association between *MTHFR* rs1801133 and the overall risk of ED. Nevertheless, the TT genotype was associated with greater ED severity, suggesting that this variant may influence disease progression rather than disease occurrence itself [23]. Our results did not demonstrate a significant association between *MTHFR* polymorphisms and baseline ED severity, although differences in study populations, ED etiology and sample size may partly explain these discrepant findings. The recently published systematic review by Ferezin et al. summarized evidence from 35 studies investigating genetic biomarkers associated with ED risk, severity and treatment response. The authors identified *MTHFR* as one of the most frequently studied genes; however, they concluded that the overall evidence remains heterogeneous and of low certainty [24]. Additionally, previous studies have shown that the effect of *MTHFR* variants on vascular outcomes may be modulated by environmental factors such as dietary folate intake and vitamin status. Moreover, interventional studies suggest that metabolic correction, rather than genotype alone, may be more relevant for improving endothelial function and erectile outcomes [22].

Another possible explanation for the lack of significant genotype–phenotype associations is the generally adequate folate status observed in our cohort. Median serum folate concentrations were within the upper part of the reference range, and no participants had folate deficiency. Relatively preserved folate status in our cohort may have attenuated phenotypic consequences of folate pathway polymorphisms and reduced the likelihood of detecting genotype–phenotype associations. This is consistent with previous observations suggesting that the clinical effects of folate-pathway variants may be modified by nutritional and metabolic factors [9]. Notably, given that plasma homocysteine was not measured, we cannot exclude that metabolic compensation, rather than a true absence of genetic effect, contributed to this negative finding; this is a central limitation of our study, discussed further below.

To date, relatively few studies have examined genetic predictors of PDE5i response. Most have focused on genes directly involved in NO signaling and vascular regulation. In their systematic review, Mostafa et al. concluded that although multiple candidate genes have shown promising associations, no pharmacogenetic marker has yet accumulated sufficient evidence for routine clinical use [7]. To our knowledge, no previous prospective study has specifically evaluated *MTHFR* and *MTR* polymorphisms in relation to tadalafil response in men with DM and ED.

A nominal association was observed between *MTR* rs1805087 and worse tadalafil treatment response in our study; this finding was based on only five GG carriers, arose in a pre-specified exploratory outcome analyzed without multiplicity adjustment, and was not reproduced with the ≥4-point responder definition, for which the global genotype test was non-significant. It should not be interpreted as a clinically relevant genotype effect, rather as hypothesis-generating evidence. The *MTR* gene encodes methionine synthase, a key enzyme responsible for the remethylation of homocysteine to methionine. Reduced enzymatic activity may lead to elevated homocysteine levels and impaired endothelial function [10]. Given that PDE5i such as tadalafil act downstream of NO production, reduced NO availability may limit their therapeutic efficacy; this remains a plausible explanation for the observed signal, but is not directly tested by our data. This concept is supported by previous studies showing that correction of hyperhomocysteinemia through folate and vitamin supplementation can improve erectile function and enhance responsiveness to PDE5i in selected patients [22].

The deviation from Hardy–Weinberg equilibrium observed for *MTRR* rs1801394 merits comment. Genotyping quality control for this SNP followed the same protocol as the other polymorphisms, including ~20% blinded duplicate genotyping with 100% concordance and known control samples on every plate (Section 2.3), which argues against a simple genotyping artifact as the sole explanation. More likely contributors include the relatively small sample size, chance, and selection of a specific disease population (men with DM and ED), any of which can produce apparent HWE deviation without reflecting a true genotyping error. An exact HWE test [25] was performed *post hoc* and confirmed the deviation (*p* = 0.006), closely matching the asymptotic chi-square result (*p* = 0.003) and indicating that the finding is not an artifact of the chi-square. Because of this deviation, *MTRR* rs1801394 was excluded from further association analyses in this study, and no conclusions could be drawn regarding its role in ED severity or tadalafil response.

Importantly, our findings do not provide sufficient evidence to support routine genotyping of these folate-pathway variants as a means of predicting tadalafil response in men with diabetes, although smaller genetic effects cannot be excluded. Clinical factors, rather than these specific genetic markers, should remain the primary basis for individualizing ED management in this population. Our study expands the current pharmacogenetic framework in ED by highlighting the potential contribution of metabolic pathways upstream of NO signaling, while most previous research has focused on genes directly involved in NO synthesis or PDE5 activity.

This study has several important limitations. First, the relatively small sample size limits statistical power, particularly for detecting modest genetic effects, which are typical in pharmacogenetic studies. The study was powered to detect only large effect sizes. Therefore, smaller but potentially clinically relevant genetic effects could not be excluded, and this limitation applies as much to the negative *MTHFR* findings as to the imprecise *MTR* rs1805087 estimate. Second, the number of individuals carrying certain genotypes was low, limiting the robustness of subgroup analyses. The nominal association observed for *MTR* rs1805087 was driven by a very small number of individuals carrying the GG genotype, resulting in wide confidence intervals and limiting the robustness of the observed effect estimate. Due to the deviation from HWE for *MTRR*, we could not assess the role of this polymorphism in our study. Third, the ≥4-point responder threshold was derived from anchor-based MCID analyses of the full IIEF erectile function domain rather than of the abbreviated IIEF-5/SHIM; no formally validated MCID exists for the IIEF-5. This extrapolation is a limitation of the responder definition used here, although a ≥4-point change remains a reasonable exploratory threshold and the direction of our findings was unchanged across the alternative ‘any improvement’ and IIEF-5 ≥ 22 definitions. Fourth, plasma homocysteine levels and vitamin B6/B12 cofactor levels were not measured, precluding direct assessment of the proposed mechanistic link between genotype, metabolic alterations, and treatment response. Although homocysteine represents the most plausible mechanistic link between folate pathway polymorphisms and endothelial dysfunction, our study was designed as a pharmacogenetic association study rather than a metabolic study and therefore plasma homocysteine concentrations were not available. As folate pathway polymorphisms are thought to act primarily through their downstream effect on homocysteine rather than through a direct effect on vascular tissue, this omission is a fundamental limitation of the mechanistic interpretation of our findings and, combined with the relatively high baseline folate levels observed in this cohort, means we cannot exclude that metabolic compensation, rather than a true absence of genetic effect, contributed to our negative results. Fifth, participants were recruited from two Slovenian centers and were of European ancestry, which limits generalizability to other populations in whom allele frequencies and folate status may differ. Sixth, information on prior PDE5i exposure and on adherence to the prescribed tadalafil regimen was not systematically collected; unmeasured variation in either could contribute to unexplained variability in treatment response that we could not account for in our models. Last but not least, as a single-arm pre–post study without a placebo or untreated comparison group, the observed increase in IIEF-5 scores over the treatment period cannot be attributed to a pharmacological effect of tadalafil with the same certainty as in a randomized, placebo-controlled design.

Despite these limitations, this study has several strengths. To our knowledge, it is among the first prospective pharmacogenetic investigations examining folate pathway polymorphisms in diabetic men receiving tadalafil therapy. The prospective design, standardized treatment regimen, and use of validated clinical outcome measures strengthen the reliability of the findings. Furthermore, the study expands current pharmacogenetic research beyond genes directly involved in NO signaling by examining metabolic pathways that may influence endothelial function upstream of PDE5 activity. This pathway-based perspective may be particularly relevant in complex conditions such as diabetic ED, where multiple pathophysiological mechanisms converge.

## 5. Conclusions

This exploratory, hypothesis-generating study of men with diabetes and ED found that common polymorphisms in folate-pathway genes do not appear to substantially influence baseline ED severity, and overall, our findings do not support a major role for common variation in these genes in tadalafil treatment response. While a nominal association was observed for *MTR* rs1805087, this was based on a very small subgroup, did not remain significant after correction for multiple testing, and did not replicate using the clinically relevant ≥4-point responder definition; it should be regarded strictly as hypothesis-generating. Overall, our findings do not provide evidence for a major effect of the investigated common variants on tadalafil treatment response in this cohort; however, smaller, potentially clinically relevant genetic effects cannot be excluded. Larger studies integrating genetic variation, folate levels, plasma homocysteine and B-vitamin cofactor status, endothelial biomarkers and clinical outcomes are needed to further clarify the role of folate metabolism in personalized management of diabetic ED. Adequately powered, multi-ethnic cohorts with direct measurement of plasma homocysteine would be particularly valuable to confirm or refute the nominal *MTR* rs1805087 signal reported here.

## Figures and Tables

**Figure 1:**
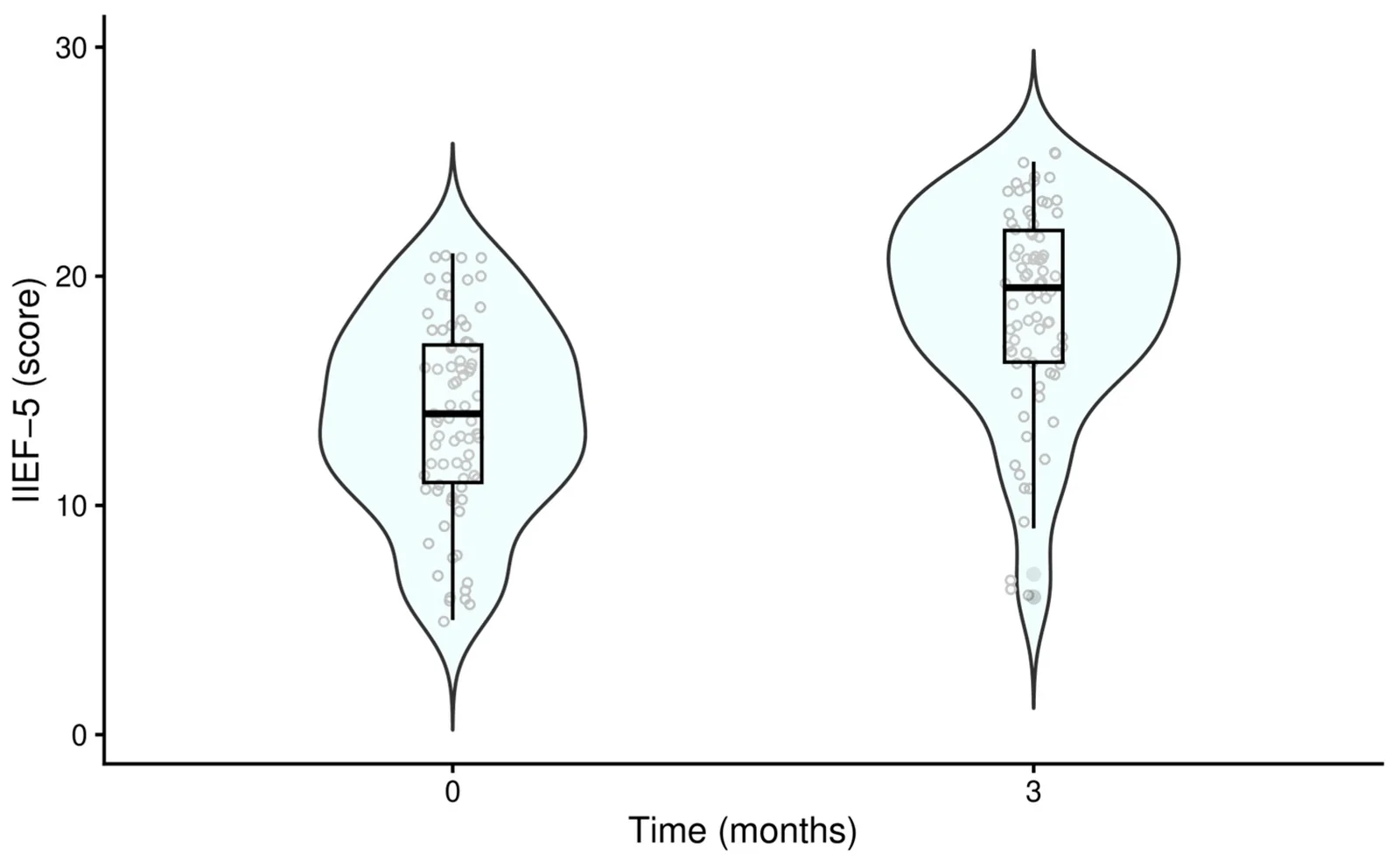
Distribution of IIEF-5 scores at baseline and after 3 months of treatment in patients with ED. Violin plots show the full score distribution at each timepoint; box plots indicate median and interquartile range; individual patient values are overlaid as points.

**Table 1:** Clinical characteristics of the included patients (*N* = 78).

Variable	Category/Unit	Statistic
Age	Years, median (IQR)	58 (53–62.3)
Duration of DM	Years, median (IQR)	11 (6–17)
HbA1c	%, median (IQR)	7.2 (6.4–8.3)
DM type	1, *n* (%)	9 (11.5)
	2, *n* (%)	69 (88.5)
Total testosterone [1]	nmol/L, median (IQR)	11.7 (8.9–17.5)
SHBG [1]	nmol/L, median (IQR)	31 (22–47)
LH [1]	IU/L, median (IQR)	4.3 (3.4–5.7)
FSH [1]	IU/L, median (IQR)	7 (5–11.2)
Prolactin [1]	μg/L, median (IQR)	5.9 (4.3–7.8)
Folates [9]	nmol/L, median (IQR)	26.4 (20.3–30.2)
TSH [1]	mIU/L, median (IQR)	2 (1.4–2.5)
T4 [1]	pmol/L, median (IQR)	15.3 (14.2–16.5)
T3 [1]	pmol/L, median (IQR)	5.2 (4.9–5.6)
PSA [1]	μg/L, median (IQR)	0.68 (0.39–1.25)

The number of missing data is given in square brackets. DM—diabetes mellitus, SHBG—sex hormone-binding globulin, LH—luteinizing hormone, FSH—follicle-stimulating hormone, TSH—thyroid-stimulating hormone, T4—thyroxine, T3—triiodothyronine, PSA—prostate-specific antigen.

**Table 2:** IIEF-5 questionnaire results.

Variable	Category/Unit	Statistic
IIEF-5 before treatment	Points, median (IQR); [min–max]	14 (11–17); [5–21]
ED severity before treatment	Mild, *n* (%)	22 (28.2)
	Mild to moderate, *n* (%)	32 (41.0)
	Moderate, *n* (%)	16 (20.5)
	Severe, *n* (%)	8 (10.3)
IIEF-5 after treatment	Points, median (IQR)	19.5 (16–22)
IIEF-5 change	Points, median (IQR); [min–max]	4 (2–7); [0–15]
IIEF-5 improvement after treatment	No, *n* (%)	15 (19.2)
	Yes, *n* (%)	63 (80.8)
IIEF-5 improvement of at least four points after treatment	No, *n* (%)	31 (39.7)
	Yes, *n* (%)	47 (60.3)

IIEF-5—International Index of Erectile Function, ED—erectile dysfunction.

**Table 3:** Genotype frequencies for the studied polymorphisms, minor allele frequencies and agreement with Hardy–Weinberg equilibrium.

Gene	Polymorphism	Genotype	*n* (%)	MAF (%)	PHWE
MTHFR	rs1801131	AA	43 (55.1)	25.0	0.597
		AC	31 (39.7)		
		CC	4 (5.1)		
	rs1801133	CC	29 (37.2)	38.5	0.797
		CT	38 (48.7)		
		TT	11 (14.1)		
MTR	rs1805087	AA	43 (55.1)	25.6	0.939
		AG	30 (38.5)		
		GG	5 (6.4)		
MTRR	rs1801394	AA	14 (17.9)	48.7	0.003
		AG	52 (66.7)		
		GG	12 (15.4)		

A—adenine, G—guanine, C—cytosine, T—thymine, MAF—minor allele frequency, P_HWE_—*p*-value of the χ^2^ test for Hardy–Weinberg equilibrium (HWE). HWE tests were treated as a separate pre-analysis quality-control family of four tests, with a Bonferroni-corrected threshold of *p* < 0.0125 (0.05/4); this family is independent of the outcome analyses (Section 2.4). Genotypes are reported on the reference (plus) strand for all polymorphisms; see Section 2.3.

**Table 4:** Folate pathway polymorphisms and change in IIEF-5.

Gene	Polymorphism	Genotype	Δ IIEF-5	*p*
MTHFR	rs1801131	AA	4 (2–7)	*p*_add_ = 0.735
		AC	4 (1–9)	
		CC	3 (0–6.8)	
		AC + CC	4 (0–8)	*p*_dom_ = 0.624
	rs1801133	CC	5 (2.5–9)	*p*_add_ = 0.456
		CT	4 (0.8–7)	
		TT	3 (2–6)	
		CT + TT	4 (1.5–7)	*p*_dom_ = 0.320
MTR	rs1805087	AA	5 (3–7)	*p*_add_ = 0.249
		AG	4 (2–7)	
		GG	0 (0–6.5)	
		AG + GG	4 (1–7)	*p*_dom_ = 0.227

Δ IIEF-5—change in IIEF-5 score from month 0 (baseline) to month 3, presented as median (IQR); *p*_add_—*p*-value for the additive model determined by the Kruskal–Wallis test; *p*_dom_—*p*-value for the dominant model determined by the Mann–Whitney test. Secondary/exploratory analysis: nominal *p*-values are reported descriptively and were not adjusted for multiple testing.

**Table 5:** Folate pathway polymorphisms and proportion of moderate or severe ED before treatment.

			ED Severity		Unadjusted OR			Adjusted OR		
Gene	SNP	Genotype	Mild/Mild-Mod	Mod/Severe	OR	95% CI	*p*	OR	95% CI	*p*
MTHFR	rs1801131	AA	32 (74.4)	11 (25.6)	Ref.			Ref.		
		AC	18 (58.1)	13 (41.9)	2.06	(0.78–5.54)	0.143	1.95	(0.73–5.31)	0.180
		CC	4 (100)	0 (0)	0.31	(0.01–3.32)	0.387	0.55	(0.01–7.20)	0.693
		AC + CC	22 (62.9)	13 (37.1)	1.70	(0.66–4.46)	0.276	1.65	(0.63–4.39)	0.310
	rs1801133	CC	22 (75.9)	7 (24.1)	Ref.			Ref.		
		CT	25 (65.8)	13 (34.2)	1.59	(0.56–4.75)	0.387	1.37	(0.46–4.22)	0.569
		TT	7 (63.6)	4 (36.4)	1.80	(0.41–7.56)	0.425	1.12	(0.21–5.32)	0.890
		CT + TT	32 (65.3)	17 (34.7)	1.62	(0.60–4.64)	0.346	1.35	(0.48–4.00)	0.570
MTR	rs1805087	AA	29 (67.4)	14 (32.6)	Ref.			Ref.		
		AG	23 (76.7)	7 (23.3)	0.65	(0.22–1.79)	0.409	0.70	(0.24–1.96)	0.497
		GG	2 (40)	3 (60)	2.85	(0.50–18.83)	0.235	1.50	(0.21–11.19)	0.675
		AG + GG	25 (71.4)	10 (28.6)	0.84	(0.32–2.17)	0.716	0.77	(0.28–2.06)	0.606

Mild/Mild-mod and Mod/Severe—mild/mild-to-moderate and moderate/severe ED categories, respectively, presented as *n* (%); Ref.—reference category; Adjusted OR—odds ratio controlling for HbA1c, age, and DM duration; ORs are Firth-penalized estimates with profile-penalized-likelihood confidence intervals; secondary/exploratory analysis: nominal *p*-values are reported descriptively and were not adjusted for multiple testing. All models included the full sample of 78 patients; no covariate data were missing.

**Table 6:** Folate pathway polymorphisms and clinically relevant improvement in ED (≥4-point increase in IIEF-5).

			Δ IIEF-5 ≥ +4 pts.		Unadjusted OR			Adjusted OR		
Gene	SNP	Genotype	No	Yes	OR	95% CI	*p*	OR	95% CI	*p*
MTHFR	rs1801131	Global					0.607			0.629
		AA	15 (34.9)	28 (65.1)	Ref.			Ref.		
		AC	14 (45.2)	17 (54.8)	0.66	(0.26–1.67)	0.375	0.65	(0.25–1.64)	0.359
		CC	2 (50.0)	2 (50.0)	0.54	(0.08–3.84)	0.523	0.41	(0.05–3.29)	0.388
	rs1801133	Global					0.226			0.293
		CC	11 (37.9)	18 (62.1)	Ref.			Ref.		
		CT	13 (34.2)	25 (65.8)	1.17	(0.43–3.17)	0.750	1.09	(0.39–3.03)	0.868
		TT	7 (63.6)	4 (36.4)	0.37	(0.09–1.45)	0.155	0.29	(0.05–1.26)	0.101
MTR	rs1805087	Global					0.311			0.402
		AA	14 (32.6)	29 (67.4)	Ref.			Ref.		
		AG	14 (46.7)	16 (53.3)	0.56	(0.21–1.44)	0.227	0.55	(0.21–1.44)	0.225
		GG	3 (60.0)	2 (40.0)	0.35	(0.05–2.01)	0.235	0.46	(0.07–2.83)	0.401

Δ IIEF-5 ≥ +4 pts.—increase of ≥4 points in IIEF-5 score from baseline (month 0) to month 3, presented as *n* (%); Ref.—reference category; Adjusted OR—odds ratio controlling for HbA1c, age, DM duration, and IIEF-5 score at baseline; ORs are Firth-penalized estimates with profile-penalized-likelihood CIs; inference for the primary endpoint is based on the global genotype test for each SNP (Section 2.4), with a Bonferroni-corrected threshold of *p* < 0.0167 (0.05/3) applied across the three tests; the genotype-specific ORs and *p*-values shown here are exploratory effect estimates and are not multiplicity-adjusted; all models included the full sample of 78 patients; no covariate data were missing.

**Table 7:** Folate pathway polymorphisms and improvement in ED after treatment (‘any improvement’ in IIEF-5).

			Δ IIEF-5 ≥ +1 pt.		Unadjusted OR			Adjusted OR		
Gene	SNP	Genotype	No	Yes	OR	95% CI	*p*	OR	95% CI	*p*
MTHFR	rs1801131	AA	6 (14)	37 (86)	Ref.			Ref.		
		AC	7 (22.6)	24 (77.4)	0.57	(0.17–1.83)	0.339	0.56	(0.17–1.77)	0.320
		CC	2 (50)	2 (50)	0.17	(0.02–1.30)	0.086	0.21	(0.02–1.73)	0.142
		AC + CC	9 (25.7)	26 (74.3)	0.48	(0.15–1.46)	0.197	0.48	(0.15–1.43)	0.190
	rs1801133	CC	5 (17.2)	24 (82.8)	Ref.			Ref.		
		CT	9 (23.7)	29 (76.3)	0.70	(0.20–2.22)	0.545	0.74	(0.21–2.46)	0.621
		TT	1 (9.1)	10 (90.9)	1.57	(0.26–16.71)	0.639	1.23	(0.19–13.34)	0.835
		CT + TT	10 (20.4)	39 (79.6)	0.84	(0.25–2.59)	0.772	0.87	(0.25–2.81)	0.822
MTR	rs1805087	AA	7 (16.3)	36 (83.7)	Ref.			Ref.		
		AG	5 (16.7)	25 (83.3)	0.95	(0.28–3.35)	0.938	0.96	(0.28–3.50)	0.953
		GG	3 (60)	2 (40)	0.15	(0.02–0.89)	0.037	0.16	(0.02–1.08)	0.060
		AG + GG	8 (22.9)	27 (77.1)	0.66	(0.22–2.01)	0.466	0.73	(0.23–2.26)	0.583

Δ IIEF-5 ≥ +1 pt.—any increase (≥1 point) in IIEF-5 score from baseline (month 0) to month 3, presented as *n* (%); Ref.—reference category; Adjusted OR—odds ratio controlling for HbA1c, age, DM duration, and IIEF-5 score at baseline; ORs are Firth-penalized estimates with profile-penalized-likelihood CIs; secondary/exploratory analysis: nominal *p*-values are reported descriptively and were not adjusted for multiple testing; all models included the full sample of 78 patients; no covariate data were missing.

**Table 8:** Baseline clinical characteristics of responders vs. non-responders (≥4-point IIEF-5 improvement).

	Δ IIEF-5 ≥ +4 pts.		
Variable	No	Yes	*p*
Age	58.0 (52.5–62.0)	58.0 (54.0–63.0)	0.841
DM duration	11.0 (7.0–17.0)	11.0 (5.5–17.0)	0.713
HbA1c	7.0 (6.2–8.1)	7.3 (6.5–8.4)	0.482
Baseline IIEF-5 score	16.0 (12.0–18.0)	13.0 (10.5–16.0)	0.099
Total testosterone ^a^	11.6 (9.0–15.0)	12.8 (8.4–18.1)	0.613
Serum folate ^b^	26.6 (20.2–31.5)	26.4 (21.6–29.8)	0.973
DM type 2	30/31 (96.8)	39/47 (83.0)	0.079
SGLT2 inhibitor use ^a^	23/30 (76.7)	29/47 (61.7)	0.264

Data presented as median (IQR) for continuous variables and *n* (%) for categorical variables (DM type 2, SGLT2 inhibitor use). Unless otherwise indicated, *n* = 31 for non-responders and *n* = 47 for responders; baseline comparisons are descriptive; nominal *p*-values are reported without adjustment for multiple testing. ^a^ *n* = 30 for total testosterone/SGLT2 inhibitor use (non-responders). ^b^ *n* = 26 for Serum folate (non-responders) and *n* = 43 (responders).

## Data Availability

The data presented in this study are available on reasonable request from the corresponding author.
